# Achieving Better Understanding of Obstructive Sleep Apnea Treatment Effects on Cardiovascular Disease Outcomes through Machine Learning Approaches: A Narrative Review

**DOI:** 10.3390/jcm13051415

**Published:** 2024-02-29

**Authors:** Oren Cohen, Vaishnavi Kundel, Philip Robson, Zainab Al-Taie, Mayte Suárez-Fariñas, Neomi A. Shah

**Affiliations:** 1 Department of Medicine, Division of Pulmonary, Critical Care and Sleep Medicine, Icahn School of Medicine at Mount Sinai, New York, NY 10029, USA; oren.cohen@mountsinai.org (O.C.); vaishnavi.kundel@mssm.edu (V.K.); 2 Biomedical Engineering and Imaging Institute, Icahn School of Medicine at Mount Sinai, New York, NY 10029, USA; philip.robson@mountsinai.org; 3 Center for Biostatistics, Department of Population Health Science and Policy, Icahn School of Medicine at Mount Sinai, New York, NY 10029, USA; zainab.al-taie@mountsinai.org (Z.A.-T.); mayte.suarezfarinas@mssm.edu (M.S.-F.)

**Keywords:** obstructive sleep apnea, cardiovascular disease, machine learning, artificial intelligence, heterogeneity of treatment effects, ethics in machine learning and artificial intelligence

## Abstract

Obstructive sleep apnea (OSA) affects almost a billion people worldwide and is associated with a myriad of adverse health outcomes. Among the most prevalent and morbid are cardiovascular diseases (CVDs). Nonetheless, randomized controlled trials (RCTs) of OSA treatment have failed to show improvements in CVD outcomes. A major limitation in our field is the lack of precision in defining OSA and specifically subgroups with the potential to benefit from therapy. Further, this has called into question the validity of using the time-honored apnea–hypopnea index as the ultimate defining criteria for OSA. Recent applications of advanced statistical methods and machine learning have brought to light a variety of OSA endotypes and phenotypes. These methods also provide an opportunity to understand the interaction between OSA and comorbid diseases for better CVD risk stratification. Lastly, machine learning and specifically heterogeneous treatment effects modeling can help uncover subgroups with differential outcomes after treatment initiation. In an era of data sharing and big data, these techniques will be at the forefront of OSA research. Advanced data science methods, such as machine-learning analyses and artificial intelligence, will improve our ability to determine the unique influence of OSA on CVD outcomes and ultimately allow us to better determine precision medicine approaches in OSA patients for CVD risk reduction. In this narrative review, we will highlight how team science via machine learning and artificial intelligence applied to existing clinical data, polysomnography, proteomics, and imaging can do just that.

## 1. Introduction

Obstructive sleep apnea (OSA) affects almost one billion people worldwide and 24 million people in the United States alone [1]. Despite the magnitude of this disorder, there remains a considerable knowledge gap in how we address its implications. The belief that all OSA patients require treatment has been questioned due to the lack of concrete evidence supporting this stance [2,3]. For instance, while continuous positive airway pressure (CPAP) enhances measures of sleepiness, blood pressure, and overall quality of life [4,5], its positive influence on cardiovascular disease (CVD) risk has not been consistently demonstrated, especially among nonsleepy OSA patients [6,7,8]. This inconsistency in outcomes suggests that the OSA population is heterogeneous and that not all patients derive equal benefits from CPAP.

Moreover, there is an absence of clinical risk-prediction tools specifically for CVD in OSA patients, though there are ongoing efforts within this domain [9,10]. Clinicians find it challenging to prioritize treatment for those at elevated risk, underscoring the need for more sophisticated, data-driven solutions. The current challenges focus on optimizing treatment plans, discerning those at increased risk of primary and recurrent CVD events, and identifying those patients who might benefit from interventions like CPAP for CVD risk mitigation. The significance of developing machine learning (ML)/artificial intelligence (AI)-based prediction tools for CVD risk reduction especially in asymptomatic OSA patients cannot be overstated. Randomized clinical trials (RCTs), unfortunately, do not always provide clarity on the full scope of treatment benefits. Though the major RCTs have not shown significant advantages of CPAP in decreasing CVD events in nonsleepy OSA patients, we speculate that this may be partially due to heterogeneity in treatment responses, i.e., not everyone with OSA will experience CVD risk reduction when CPAP is applied. 

ML and AI present promising avenues for advancing our understanding and treatment of OSA. Both the National Institutes of Health (NIH) and the American Heart Association (AHA) have recognized the potential of ML/AI for advancing our understanding of how sleep disorders impact cardiovascular health and the need for fine-tuning treatment personalization [11,12]. Incorporating ML into medical research has led to the discovery of novel causal contributors to adverse outcomes [13,14]. For example, in the PARADIGM registry, ML models outperformed conventional statistical models and atherosclerotic CVD risk scores in identifying individuals at risk of rapid progression of coronary atherosclerosis [15]. In the Multi-Ethnic Study of Atherosclerosis (MESA), ML more accurately predicted the CVD event rate compared to traditional risk scores [16]. The heterogeneity of OSA disease presentations, risk factors, overlapping comorbidities, and treatment outcomes make it an ideal condition for the application of ML/AI. Vast clinical, biomedical, and polysomnographic information in OSA patients often remains underutilized in current analyses due to the magnitude of and interdependencies within the data. There has been significant interest and progress in employing ML/AI to develop more effective diagnostic and monitoring programs for OSA. However, striking the right balance between ideal methods and practical constraints is essential in this pursuit. Furthermore, the application of ML in sleep medicine opens new avenues of investigation into the issue of treatment heterogeneity. Current projects within our group are applying ML to RCT data to develop advanced decision tools to identify nonsleepy OSA patient subgroups with differential treatment responses, a task that has been challenging using traditional methodologies. Furthermore, new advanced AI technologies, such as transformer-based neural networks, can augment ML-based applications. Transformers can effectively process raw image data, such as computed tomography (CT) scans of the face, oral cavity, or chest, or polysomnographic (PSG) data, enabling the automated recognition and categorization of prevalent sleep apnea-related patterns.

However, a major hurdle of ML/AI is the “black box” phenomenon, where the process from input to outcome remains obscured. This opaqueness can deter trust in the system, particularly for clinicians, researchers, and educators unfamiliar with ML/AI and its strengths and weaknesses. Thoroughly evaluating the output of ML is just as crucial as crafting the model itself, especially when considering its potential integration into clinical practice. Addressing this requires a team-science approach, blending the expertise of clinicians, data scientists, statisticians, and clinical bioinformaticians. The objective is to produce robust, high-performance prediction models that can be readily translated into clinical practice.

This review will focus on the crucial role of ML/AI in achieving a more patient-centered diagnosis of OSA by replacing traditional diagnostic metrics, as well as its application in understanding treatment heterogeneity. We believe that harnessing state-of-the-art ML/AI techniques to analyze extensive OSA datasets will usher in the long-awaited era of personalized medicine for OSA. 

## 2. Statistical Methodology and Machine Learning Algorithms

ML, a subset of AI, uses computer algorithms to identify complex interactions in large, multidimensional datasets that might elude human analysis. There are currently three important forms of ML/AI currently in use: supervised, unsupervised, and reinforcement learning frameworks. Supervised approaches are characterized by their ability to learn the underlying relationships between predictor variables and known outcomes [17]. These types of analyses can be used in medicine for risk assessment, diagnosis, and predicting treatment outcomes. This category includes both models that are intuitive and easy to interpret, such as linear models and decision trees, as well as those that capture more complex interactions between predictor and outcome variables. Simpler models offer intuitive and more easily interpretable predictions based on patient features, but they often lack robustness and are sensitive to random perturbations in the data. More complex algorithms, including the support vector machine, random forest, and deep learning, can be very powerful and robust but difficult to understand. For example, a random forest model built using data from the Sleep Heart Health Study was more accurate in predicting 10-year CVD risk than the Framingham Risk Score (FRS) [18]. However, it is not possible to understand all the ways in which variables within the model interact to produce its predictions. Therefore, using such a model, it is not easy to identify specific clinical features to target for intervention. Users can only feed a patient’s data into the model to obtain a risk prediction. Conversely, for the FRS, each variable and its weighted importance (i.e., the number of points it contributes to the score) are published. Thus, a clinician can assess which variables from an individual patient’s history are contributing to a given score and tally their score manually. An ensemble method such as random forests improves the algorithm accuracy by stabilizing the model performance through averaging the outcomes of multiple decision trees. However, explaining the interaction between features within the model can still be problematic. Lastly, even more advanced methods like survival forests use similar ensemble methods but focus on time-to-event data, providing a measure of risk over time [19].

Unsupervised learning attempts to learn patterns from seemingly random data within large datasets [20]. This takes on two main forms: (1) the clustering of participants based on underlying data and (2) dimensionality reduction, which uncovers a smaller number of hidden features that best represent and summarize the data without loss of information. Clustering methods are useful for identifying disease phenotypes and subgroups with similar characteristics, and have evolved from commonly used methods, such as latent class analysis (LCA), hierarchical clustering, and K-means clustering, to more advanced deep learning-based approaches. A study by Bailly et al. represents a prime example of clustering within OSA utilizing multiple data domains, including clinical and PSG data, from a large European database [21]. Using LCA, the authors found eight distinct phenotypes among 23,139 OSA patients. Further, they found that the rate of CPAP prescription varied between groups, with overweight men and women having some of the lowest prescription rates (57% and 49%, respectively), while younger/sleepier obese patients as well as older obese men had the highest rates of CPAP prescription (94% and 93%, respectively). The second category of unsupervised learning, dimensionality reduction, includes methods such as the linear principal component analysis (PCA), the nonlinear uniform manifold approximation and projection (UMAP), and more complex deep learning-based autoencoders. These types of analyses are useful for identifying a parsimonious list of features that represent the data while reducing the redundancy present with other methods, such as clustering. PSG is a great example of high-dimensional data capturing a multitude of physiologic signals. Dimensionality reduction techniques allow us to combine and pare down this information. For example, PCA can be applied to clinical [22] and/or PSG [23] data to reduce the number of features, and combined with clustering methods to identify unique patient subgroups from immense streams of data, which would be missed by conventional clinical scores and traditional PSG criteria.

While both the aforementioned supervised and unsupervised ML methods are static, reinforcement learning is a dynamic learning method by which an algorithm continues to evolve using feedback from past experiences to improve its performance [24]. Autotitrating CPAP is an excellent candidate for the application of reinforcement learning. Currently, autotitrating CPAP responds to flow limitation from a fixed scanning window. However, using the principles of reinforcement learning, it is possible to develop a system that could also learn patterns within or across nights to better optimize continuous pressure adjustment adapted to an individual patient in a certain position at a given time during sleep. This technique has not been utilized as much within sleep medicine as yet. However, there is great potential for its application in the future.

Lastly, though not a separate category of ML, transfer learning is another technique that must be mentioned and can be used in combination with any of the above forms of ML. Transfer learning is the application of models designed for one task/environment to another, enabling more rapid development of ML in a new setting [25,26]. As AI continues to advance, existing ML/AI models trained on large comprehensive datasets offer researchers and clinicians a strong foundation for creating new, more specialized, and effective diagnostic and treatment decision tools in other datasets. For example, a supervised learning model that was developed in a large clinical cohort predominantly comprised of white males may perform poorly if applied directly to other cohorts. Transfer learning allows us to retrain and adjust this existing model in a cohort that has a higher percentage of women and racial/ethnic diversity. Transfer learning can also improve ML performance when applied to smaller cohorts in a phenotypic subgroup; for example, as it uses the robust model initially developed within a larger population but tailored to this subgroup. Not only does transfer learning accelerate the development process, but it also produces more precise and efficient solutions, particularly in these types of data-limited scenarios.

## 3. Applying Machine Learning and Artificial Intelligence to Obstructive Sleep Apnea Data Domains

### 3.1. Assessment of Clinical Data

While the power of ML really shines when analyzing high-dimensional data, such as PSG, or handling multiple large data streams, it can still be useful in developing risk-prediction tools based solely on clinical data. Holfinger et al. used several supervised learning approaches, including the support vector machine, random forest, and artificial neural networks, to predict OSA diagnosis within the clinic-based SAGIC and Sleep Heart Health Cohorts using only age, sex, BMI, and race [27]. The authors were able to show better performance than a logistic regression model and similar performance to the STOP-BANG score, which requires more features. We must pause here, though, to highlight that the use of race in such models must be performed cautiously. Further, both cohorts used within these ML algorithms had very limited numbers of some historically and persistently excluded racial and ethnic groups. This issue of bias and ethics in ML/AI will be discussed further in a later section. Unsupervised ML approaches have also been helpful in the clinical domain. Mazzotti et al. used LCA to uncover four unique symptom phenotypes within OSA patients: “disturbed sleep”, “minimally symptomatic”, “moderately sleepy”, and “excessively sleepy” [28]. Survival analysis within these groups identified the “excessively sleepy” phenotype as having the highest risk of incident CVD. A similar analysis using LCA was performed within the Icelandic Sleep Apnoea Cohort, finding clusters with “disturbed sleep”, “minimally symptomatic”, and “excessive daytime sleepiness” [29]. These examples highlight the ways in which both supervised and unsupervised ML approaches can predict risk using a parsimonious list of features and uncover hidden relationships within clinical data.

### 3.2. Harnessing the Power of Polysomnography

The field of OSA is undergoing a paradigm shift [30,31]. Over the last several decades physicians and researchers have predominantly focused on the AHI as the primary, and often sole, measure of OSA severity, attempting to understand clinical outcomes based on this metric alone [32]. However, the AHI is a hypothesis-driven measure of OSA severity [33], developed with only the underlying disease process in mind and without consideration for disease effects on relevant outcomes [34]. This process of disease classification and study without the consideration of broader disease implications is outdated, lacking in patient-centeredness [35], and contributes to overdiagnosis. Though the AHI has been useful, it has ultimately reached its limits.

In the new age of OSA precision medicine and data-driven science, novel metrics to grade the disease severity and subtype using an individualized patient-centered approach have gained a foothold [36,37]. Four major OSA endotypes have been developed and described, including pharyngeal collapsibility, loop gain, arousal threshold, and airway dilator muscle compensation [38,39]. These distinct endotypes have been shown to be scalable using cloud-based algorithms [40] and can be used as relevant features within ML-based decision trees for personalized treatment selection [41,42,43]. There are emerging data demonstrating their utility in assessing favorability for alternative therapies such as hypoglossal nerve stimulation [44] and even blood pressure response to the CPAP treatment of OSA [45]. However, these endotypes were developed to better characterize the physiology underlying OSA-related respiratory events, not clinical outcomes such as symptoms or CVD risk.

To better understand OSA in the context of patient-centered outcomes, we can apply novel mathematical methods to identify the physiologic and clinical consequences of OSA-related respiratory events across an entire night and even breath-by-breath [46,47]. Physiologic responses to OSA events can be divided into several separate, though interconnected, axes, including arousal, sympathetic, hypoxemic, and ventilatory. Using these responses, we can better predict CVD morbidity and mortality [48,49,50]. Further, novel measures such as the pulse rate response—a surrogate for sympathetic tone—have been shown to predict CVD benefit after OSA treatment [51]. The automation of these measures will allow for greater application and combination of these features with additional clinical variables within ML models to better predict disease outcomes and treatment response.

As mentioned above, PSG data are an excellent candidate for ML/AI applications given the high dimensionality and multiple data signals. Automated scoring algorithms for PSG developed in the last two decades have shown promise in replacing manual scoring [52]. Deep-learning techniques like neural networks have been used to detect apneas and hypopneas in real time during PSG [53]. Further, the detection of these events can be achieved even in pared-down PSG signal data, such as a single respiratory channel [54] or limited EEG [55]. Layering automated analysis of physiologic responses on top of existing automation of traditional scoring will allow for a deeper understanding of patient-level data and the identification of additional features that contribute to meaningful disease outcomes in future research. For example, neural networks can meaningfully predict patient-relevant outcomes, such as daytime sleepiness [56]. Going beyond OSA itself, the data contained within a PSG and processed via neural networks can predict mortality, with much of the risk attributable to sleep fragmentation [57]. Though, it would seem from recent data that OSA-event-related arousals alone do not provide additional information regarding incident CVD [58]. Thus, there is a wealth of information available within the PSG. By applying modern machine- and deep-learning approaches to analyze PSG signals, we may finally be able to understand the complex links between OSA and health outcomes, such as CVD. Further, these robust analytical processes may allow for the more accurate assessment of OSA from wearable devices in the home sleep testing arena.

### 3.3. Proteomics to Predict Cardiovascular Disease Risk in OSA

While there have been significant advances in the physiologic endotyping and clinical phenotyping of OSA, the pathobiological mechanisms underlying OSA morbidity remain elusive. Gaining a comprehensive understanding of how physiological processes associated with respiratory events result in biological responses leading to cardiometabolic and neurocognitive dysfunction is key [59]. To better understand this disease complexity, it is important to elucidate molecular and proteomic biomarkers contributing to the basic mechanisms underlying OSA. Proteomics refers to the set of ‘big data’ technologies applied to discover protein biomarkers associated various disease states. Such analyses can be performed using a “shotgun” approach to identify all measured proteins/metabolites, or using a targeted approach centered on a group of proteins [60]. Either way, the combination of “omics”-based strategies and ML methods have the potential to revolutionize sleep medicine and boost our understanding of pathobiological pathways in OSA.

Advanced immunoassays, such as the Olink^®^ inflammation and CVD biomarker panels (Olink^®^ Bioscience, Uppsala, Sweden), allow for the exploration of personalized immunophenotyping. Olink^®^ is a proteomics array that measures plasma biomarkers reflecting inflammation, immune response, cell adhesion, and tissue remodeling using a proximity extension assay. The Olink^®^ platform has been used in several studies to identify proteins associated with various CVDs [61,62,63]. This panel has also been used in OSA patients to identify subgroups based on differential inflammatory protein expression. For example, in a recent post hoc analysis of the ISAACC study [8], Zapater et al. analyzed the proteomic profiles in 86 OSA patients admitted for acute coronary syndrome, divided into those with and without recurrent CVD events [64]. Using a supervised random forest algorithm to select relevant proteins and generate a predictive model of recurrent CVD, the authors identified 38 (of 276) cardiovascular and inflammatory proteins that were differentially expressed between the two groups. Additionally, 12 proteins emerged as predictive biomarkers, of which 3 were identified as having the highest contribution to prediction of recurrent CVD events among this cohort of OSA patients. These proteins included CXCL16, STK4, and TFPI, which are implicated in cell proliferation, communication and apoptosis, and regulation/response to inflammation and immune systems. Another study used the same proteomics panel to investigate the association between OSA severity and changes in inflammatory protein expression profiles in a cohort of women [65]. There was no significant association between OSA and protein expression after adjusting for age and BMI, though severe OSA during rapid eye movement (REM) sleep was negatively associated with Axin 1 (a protein involved in tumor suppression/regulation [66]). Severe REM OSA was also associated with reductions in Sirtuin-2 (a protein involved in metabolic regulation and adipogenesis inhibition [67,68]). In a subsequent study among men by Ljunggren et al., this REM OSA effect was not observed [69]. However, among men, an oxygen desaturation index ≥30 was associated with increased plasma levels of eight inflammatory proteins, including interferon gamma and angiotensin-converting enzyme 2.

Kundel et al. recently used unsupervised analyses to uncover three unique clusters of OSA in 46 patients with low, intermediate, and high inflammatory protein expression using the Olink^®^ panel [70]. In an exploratory analysis, the authors found a differential response to CPAP among the three clusters, with an increase in inflammatory protein expression in the “low inflammatory” cluster and a decrease in inflammatory protein expression in the “high inflammatory” cluster following three months of CPAP. Although the samples sizes were small (total n = 46), the results are hypothesis-generating, and may guide future studies in the pursuit of characterizing “at-risk” subgroups of OSA patients. A similar approach using the Olink^®^ panel was applied to nasal lavage samples collected from patients with OSA before and after initiating CPAP. In this study, Cohen et al. identified 13 proteins that significantly decreased after CPAP in a subset of participants classified as having a high baseline inflammatory protein expression by unsupervised clustering methods [71]. Many of these proteins (e.g., MCP-4, OSM, LAP TGF-beta1, and VEGF-alpha) have been linked to immune cell differentiation, chemotaxis, airway inflammation, and vascular remodeling. Further validation of these results using a combination of omics with ML algorithms can help risk-stratify OSA patients for future clinical trials for CVD risk reduction.

Despite these advances, there remains a long road ahead in identifying reliable OSA biomarkers from the extensive array of options offered by proteomics. Sleep medicine has traditionally lagged behind in the integration of omics data, leaving a significant knowledge gap in our understanding of sleep disorders like OSA. The emerging landscape of OSA’s association with inflammation and CVD risk demands a more comprehensive approach. Integrating ML and omics data can unlock crucial insights into the molecular underpinnings of OSA and its impact on CVD risk. Moreover, embracing unsupervised ML approaches will be imperative for uncovering novel biomarkers that may have been previously overlooked. By combining ML/AI and omics, we have the potential to revolutionize sleep research, allowing us to (1) identify distinct subgroups within OSA populations with or without an elevated CVD risk, and (2) monitor OSA treatment efficacy [72]. This holistic approach can pave the way for more personalized diagnostics and treatments in sleep medicine.

### 3.4. Image-Based Machine Learning in OSA

Multiple ML approaches can be applied to OSA-related imaging data. Morphometric analysis, used in facial recognition technology, employs ML to analyze distances and arrangements of facial landmarks. Researchers have used the morphometric analysis of facial landmarks based on both 2D [73] and 3D [74,75] photography to differentiate between those with and without OSA. In a similar approach, Tsuiki et al. [76] used AI to analyze oropharynx architectures on 2D cephalometric radiographs. Automation of these technologies will allow for anatomic phenotyping within OSA and has the potential to replace OSA screening or even traditional sleep studies for diagnosis.

One of the most frequently used image-based ML applications is the automated segmentation of anatomical structures or the automatic classification of an image into different representative groups (e.g., disease versus no disease). This application typically utilizes a convolutional neural network approach. This approach allows rapid and robust segmentation to facilitate the measurement of dimensions and volumes of anatomical structures of interest. Extracted metrics can subsequently be used in diagnostic decision trees or in risk stratification. In the setting of OSA, most attention has been given to segmenting features of the upper airway based on 3D CT and magnetic resonance imaging (MRI) data. Craniofacial and upper-airway morphometric features on CT imaging, including the upper airway length, the A point–nasion–B point (ANB) angle, and the gonion–gnathion–hyoid angle, have been associated with elevated CVD risk [77]. De Bataille et al. [78] and Shujaat et al. [79] have used ML in cone-beam CT imaging to measure airway volume. A number of groups have demonstrated the use of the ML-based analysis of MRI to automatically segment upper-airway structures, including the pharynx, tongue, and soft palate, that may facilitate large-scale epidemiological analyses in OSA patients in the future [80,81,82]. Molnar et al. [83] used an AI analysis based on pharyngeal adipose tissue thickness derived from MRI, sex, and neck and waist circumference to separate patients with airway obstruction from those without. In a novel approach to airway measures, ML-supported computational fluid dynamics analysis has been used to predict OSA-related airflows [84].

Image-based ML has also been applied to brain MRI scans in OSA patients. Pang et al. [85] used the support vector machine and random forest to accurately classify OSA based on diffusion tensor MRI scans of the brain. In another study, Liu et al. [86] used ML analysis of resting-state functional MRI (rs-fMRI) scans of the brain to identify OSA patients with and without cognitive impairment. Similarly, Shu et al. [87] used rs-fMRI and ML analyses to investigate cognitive impairment in OSA. Agarwal et al. [88] used a convolutional neural network analysis of brain MRI scans to predict whether OSA patients treated with CPAP would experience a negative neurological condition post-treatment. These studies highlight the potential for the image-based analysis of the related and downstream effects of OSA in a multiorgan setting. Similar ML-enabled analyses combining multimodality data may be applicable in the setting of assessing the relationships between OSA and CVD risk.

In an alternative image-based ML approach, radiomics analyzes the relationships between the intensities of spatially correlated pixels. Radiomic metrics can provide insight into subtle features, patterns, or textures in the image that may not be apparent to the human observer. Using the feature tracking—a form of radiomic analysis—of cardiac-phase MRI images, Li et al. [89] assessed left ventricular (LV) parameters among patients with OSA and controls. The authors found that OSA was associated with a higher LV mass index and indexed cellular volume of the myocardium, suggesting cellular hypertrophy. Currently, however, there is a paucity of studies utilizing image-based ML or radiomics to evaluate the impact of OSA on CVD and future CVD risk. This is despite the extensive use of cardiovascular imaging in patients with OSA. Given the potential of image-based ML in diagnosing and characterizing OSA, there is an untapped opportunity in leveraging existing imaging data, such as chest X-rays, coronary artery calcium imaging, and cardiac MRI, through AI-driven analyses. For example, by applying AI to this wealth of historical data, we could gain insights into how various treatments for OSA impact cardiovascular health. This approach could help refine treatment strategies and identify the most effective interventions for individual patients, ultimately reducing the risk of cardiovascular complications associated with OSA.

### 3.5. Adding Multiple Domains for Better Prediction

Though each data domain alone provides a considerable substrate for novel statistical and AI methodologies, the true capabilities of ML lie in its ability to combine information from multiple domains. Within the field of OSA, this includes not only PSG and information from wearable technologies, but also demographic, social, behavioral, clinical, biological, and imaging data. As discussed above, techniques such as random forest [90] have been developed to handle these tasks and remain among the most powerful analytical tools available [91]. Data-driven random forest-built prediction models using a multitude of data outperform older hypothesis-driven risk scores, such as the FRS for predicting cardiovascular outcomes [18]. Wallace et al. applied random forest techniques to multidimensional data, including sleep data, and were able to demonstrate the accurate prediction of 15-year mortality risk [92]. Though the strength of this tool is the unique integration of data, individual variable importance analyses can be performed to better understand which specific features drive an algorithm’s predictions. For example, in this aforementioned study by Wallace et al., sleep efficiency on PSG and time with oxygen saturation less than 90% were among the most important isolated features. However, demographic and comorbid health domains as whole categories were even more predictive of mortality than sleep domains. As shown, ML/AI approaches represent a new frontier in risk prediction. These promising tools combining multiple data streams will allow us to finally manage, assess, and leverage the immense information available for OSA patients.

## 4. Future Perspectives: Understanding Cardiovascular Disease Outcomes after OSA Treatment—A Futuristic Approach Using Machine Learning

Harnessing ML/AI for personalized treatment in medicine, and particularly OSA, will be a game-changer for tailoring therapy to individual needs. As described above, one common ML/AI strategy involves using supervised approaches to estimate the likelihood of a particular health outcome. This estimation can help prioritize individuals who are at higher risk. This is particularly effective in medical settings, where preventative treatments can be implemented to mitigate these risks. However, it is important to note that being at high risk does not always translate to significant benefits from a treatment. Although understanding CVD risk in OSA patients may enhance the outcomes and adherence of CPAP therapy [93], these risk assessment approaches do not directly measure how treatment changes that risk. Imagine a scenario where two patients with OSA of similar severity and symptom profile receive the same treatment (e.g., CPAP). One patient experiences a remarkable improvement in symptoms and a reduction in CVD risk, while the other sees little to no improvement in sleepiness and no risk reduction in CVD. We currently have few tools to understand and address such variations in outcomes.

Traditionally, healthcare determinations are based on the average treatment effect (ATE) of an intervention derived from large high-quality RCTs comparing the intervention to a control. However, these ATEs estimate the intervention’s effect for a hypothetical chimeric patient who is an amalgamation of all the unique participants within a study [94]. These ATEs do not fully encompass the range of patient differences and risk levels essential for pinpointing those most in need of a particular intervention or potentially others that may be harmed by an intervention. The central challenge in advancing precision medicine lies in transcending the mere estimation of ATEs and risk stratification to recognize the diversity of therapeutic responses to an intervention based on factors such as patient attributes, inherent risk, and treatment susceptibility [95]. Overcoming such challenges requires a change in paradigm. Instead of directly predicting treatment outcomes, we need to understand and identify the patterns defining the heterogeneity in patient responses to a given intervention. This shift in focus is vital for more personalized and effective healthcare decisions.

Emerging methods combining AI models and causal inference have been developed to identify patients where treatment modifies the outcome risk [96]. These methods, collectively termed heterogeneous treatment effect (HTE) analyses, measure the difference in potential outcomes for an individual if they were treated versus if they were not treated. Traditionally, RCTs include subgroup analysis to understand diverse therapeutic responses by iteratively focusing on specific variables. In the realm of OSA, classic examples include secondary analysis by disease severity or by CPAP adherence thresholds. However, this can lead to erroneous conclusions either due to multiple statistical testing or limited statistical power, particularly when subgroup samples are small [97]. HTE analyses represent a considerable methodological step forward by assessing conditional average treatment effects (CATEs). CATEs reveal the treatment effect for ML/HTE model-derived subgroups—or even individuals depending on the specific form of HTE analysis—contingent on baseline covariates.

The first methods to address HTE in the context of RCTs utilized model-based recursive partitioning (MBRP) [98,99]. MBRP combines decision trees with classical statistical models to address the heterogeneity in patient responses. MBPR starts by fitting a statistical model (e.g., logistic regression in the case of binary outcomes or the Cox model for time-to-event outcomes) with treatment as a covariate on a complete dataset. It then identifies the baseline covariate that most strongly modifies the treatment effect (i.e., has an interaction with treatment) and uses that feature to partition the population into two subgroups. The procedure is applied recursively within each subgroup until no promising variables by which to split participants are left, resulting in a decision tree. Ultimately, the product of this model-derived decision tree is discrete patient subgroups clustered by their differential responses to treatment. Further, inspection of the key factors identified as the tree’s nodes (i.e., the variables used to partition subgroups at each decision point) may lead to new and previously unfathomable hypotheses regarding associated conditions and disease mechanisms. Our group is using MBRP in ongoing work to identify the effect of CPAP on CVD outcomes among OSA participants within large RCTs.

As previously described, though supervised ML decision trees are easily interpretable, they suffer from issues of overfitting and sensitivity to noise. For purposes of risk prediction, these decision trees were expanded into random forest models [90], which combine data from a multitude of trees to improve the model accuracy. Similarly in the realm of HTEs, decision tree-based HTE methods, like MBRP, have been developed into the MBRP forest [100] and causal forest [101,102]. These methods maintain the core framework of the random forest, including recursive partitioning, subsampling, and random splits. However, they are adapted to HTEs, maximizing the ability to predict the variability of treatment effects rather than model accuracy, as is performed for risk prediction in supervised learning. These algorithms provide the foundation for developing more accurate and personalized treatment strategies, moving beyond mere associations to uncover the underlying causality.

Causal forest models also have an added benefit over decision tree-based HTE models, as they directly estimate the individual treatment effect (ITE). The ITE can then be used to create a prioritization rule ranking patients by their predicted treatment response on a continuum from potential harm to benefit. The ITE is therefore much more granular than the CATE produced by decision tree-based HTE methods, which estimates the treatment effect for a subgroup of patients [103,104]. These innovative methods go beyond mere risk prediction; they measure the potential outcomes for an individual as if they were treated compared to as if they were not. These mathematical manipulations essentially allow for the equivalent of an RCT analysis within each individual participant, despite that given individual not actually having received both an intervention and control. This level of precision represents a substantial evolution in ML applications within healthcare. The game-changer here is our newfound ability to identify precisely which patients will benefit most from a particular treatment, and to therefore prioritize patients for interventions based on their individual predicted treatment response. These tools will finally enable us to tailor treatment plans to each patient’s unique needs and maximize the likelihood of successful outcomes.

Lastly, just as transfer learning can be applied to other forms of ML, it can also be used to broaden the generalizability of HTE models trained on RCT cohorts by transferring these models and retraining them in observational datasets. This technique has the power to balance the precise causal estimates obtained in ideal RCTs and apply them to a larger number of patients in a more pragmatic setting. Although this application is in its nascency in sleep medicine, it has great potential in the near term. Further, federated learning approaches orchestrate the training of several local models from heterogeneous datasets without the need for individual participant-level data integration [105]. This form of model integration abides by local privacy laws and protects participant data while maximizing the power of large datasets [106]. This method protects study participants while allowing for global collaboration, and creates better diversity, equity, and inclusion of populations from previously under-represented countries.

In summary, the progression of HTE methods from basic ML to advanced causal forest methods and ITEs will allow the field to craft increasingly personalized and effective treatment plans for patients with OSA with relevant outcomes in mind, including improving cardiovascular outcomes. These approaches can be utilized to weigh various treatment options and their specific impact on cardiovascular health, ensuring that patients receive the most suitable interventions based on their unique characteristics and expected treatment effects. These examples highlight the potential of ML applications in healthcare to enhance patient care and cardiovascular well-being in individuals with OSA, ushering in an era of truly personalized medicine, where the right treatment is administered to the right patient at the right time. Future studies should focus on integrating these innovative approaches to fully leverage the capability of ML/AI and advanced statistical methods, making individualized treatment the norm rather than the exception.

## 5. Ethics in Machine Learning and Artificial Intelligence

Ethics in AI is a deeply important and continually evolving domain of study and discourse. As AI systems become more integrated in healthcare, the ethical implications of their applications grow in magnitude. Key issues like safety, fairness, privacy, and accountability demand action from AI developers, healthcare entities, governments, and society at large.

Bias and fairness are prominent concerns in AI, as decision-making models can inadvertently reflect and amplify societal biases in their training datasets. Research disparities persist in sleep medicine, particularly in regards to race/ethnicity, socioeconomic factors, and gender. OSA research has historically centered on males due to their higher condition prevalence, especially using older AHI criteria. This tendency is further exacerbated by sex differences in symptom presentation, driving underdiagnosis in women. Such disparities in research translate to the under-representation of certain groups in the data collected and used to train AI models. Prior research has already shown that only a minority of sleep clinic patients with OSA would meet the criteria for the existing RCTs within our field including a large proportion of women [107]. When predicting CVD risk in OSA patients using ML, such nonrepresentative training data can hinder the equity of AI models’ performance across diverse backgrounds. This could inadvertently prioritize or neglect certain demographics in risk assessments. For example, if training data lean heavily towards male OSA patients, the AI might be less accurate in assessing CVD risks for female patients due to different symptom presentations. Other biases, such as those pertaining to the variability of treatment and diagnostic criteria, must also be carefully considered. For example, patients may be on different treatments for OSA that can affect CVD risk. Further, the criteria and modality used to diagnose OSA and measure its severity might change over time or vary between institutions. Models that do not account for such heterogeneity and unbalances can inject bias into AI predictions.

To ensure fairness in AI determinations, biases must be audited and addressed before the deployment of AI models. It is crucial to train AI users and developers on the use of fairness toolkits, such AI Fairness [108]. Prospectively, the research community should ensure diversity, equity, and inclusion in studies and trials to mitigate upstream biases within clinical datasets used by AI. Biased AI predictions can result in serious ramifications, leading to either neglect or excessive medical interventions. Hence, consistent evaluation and recalibration of AI models are vital to maintain fairness and adapt to the evolving medical understanding of OSA and CVD outcomes.

Finally, complex AI models, especially in deep learning, often act as a “black box”, obscuring their decision-making and hindering trust. For patients and clinicians impacted by these models, understanding AI-driven decisions is paramount, even if it compromises peak model efficiency. The AI community is advancing and standardizing “explainable AI” [109] techniques, introducing methods like SHapley Additive exPlanation (SHAP), Local Interpretable Model-agnostic Explanations (LIME), attention mechanisms, and visualization tools. These techniques help us to understand how AI is using input data to reach its decisions and predictions. The evolution of explainable AI demands domain-specific insights for distinct needs, emphasizing the crucial role of team science with representation from both the OSA research community and data science developers. Further, the integration of AI into society requires a multidisciplinary approach, involving not just computer scientists, but also ethicists, sociologists, psychologists, and policymakers. As AI continues to advance, its users must prioritize ethical considerations to ensure that the technology benefits humanity and does not inadvertently harm or disadvantage certain groups.

## 6. Conclusions

In conclusion, ML and AI are important tools that have been used and developed in many fields of science and medicine. Their use in OSA is particularly exciting given the emerging research in our field uncovering disease heterogeneity and variability in treatment effects. The myriad of physiologic, biologic, and clinical data available for patients with OSA in the digital age from electronic health records, PSG, imaging, and multiomics are ripe for data science techniques that can combine multiple domains and assess high-dimensional data to improve patient experience, risk prediction, and treatment outcomes. Advances within ML/AI will allow for more complex analyses tailored to answer specific research questions and generate hypotheses previously unfathomable. However, despite the enticing features of ML/AI, we must remain cautious and vigilant to not overstep or introduce bias, as these methods have the potential to worsen pre-existing disparities in sleep medicine. OSA researchers using these methodologies must be rigorous and uncompromising on quality and fairness. We hope that the application of ML/AI will not only help identify patients who will benefit from OSA treatment, but also those who may potentially be harmed, aligning with the principles of the Hippocratic oath.

## Data Availability

Not applicable.
